# Exploring the role and therapeutic potential of lipid metabolism in acute kidney injury

**DOI:** 10.1080/0886022X.2024.2403652

**Published:** 2024-09-25

**Authors:** Xiaoyu Zhang, Wen Wu, Yiming Li, Zhiyong Peng

**Affiliations:** a Department of Critical Care Medicine, Zhongnan Hospital, Wuhan University, Wuhan, China; b Clinical Research Center of Hubei Critical Care Medicine, Wuhan, China; c Department of Critical Care Medicine, Yichang Central People’s Hospital, Yichang, China; d Department of Critical Care Medicine, Center of Critical Care Nephrology, University of Pittsburgh School of Medicine, Pittsburgh, PA, USA

**Keywords:** Acute kidney injury, lipid, metabolism, FAO, clinical translation

## Abstract

Acute kidney injury (AKI) is a prevalent condition, yet no specific treatment is available. Extensive research has revealed the pivotal role of lipid-related alterations in AKI. Lipid metabolism plays an essential role in the sustenance of the kidneys. In addition to their energy-supplying function, lipids contribute to the formation of renal biomembranes and the establishment of the renal microenvironment. Moreover, lipids or their metabolites actively participate in signal transduction, which governs various vital biological processes, such as proliferation, differentiation, apoptosis, autophagy, and epithelial–mesenchymal transition. While previous studies have focused predominantly on abnormalities in lipid metabolism in chronic kidney disease, this review focuses on lipid metabolism anomalies in AKI. We explore the significance of lipid metabolism products as potential biomarkers for the early diagnosis and classification of AKI. Additionally, this review assesses current preclinical investigations on the modulation of lipid metabolism in the progression of AKI. Finally, on the basis of existing research, this review proposes future directions, highlights challenges, and presents novel targets and innovative ideas for the treatment of and intervention in AKI.

## Visual Abstract

**Figure:**
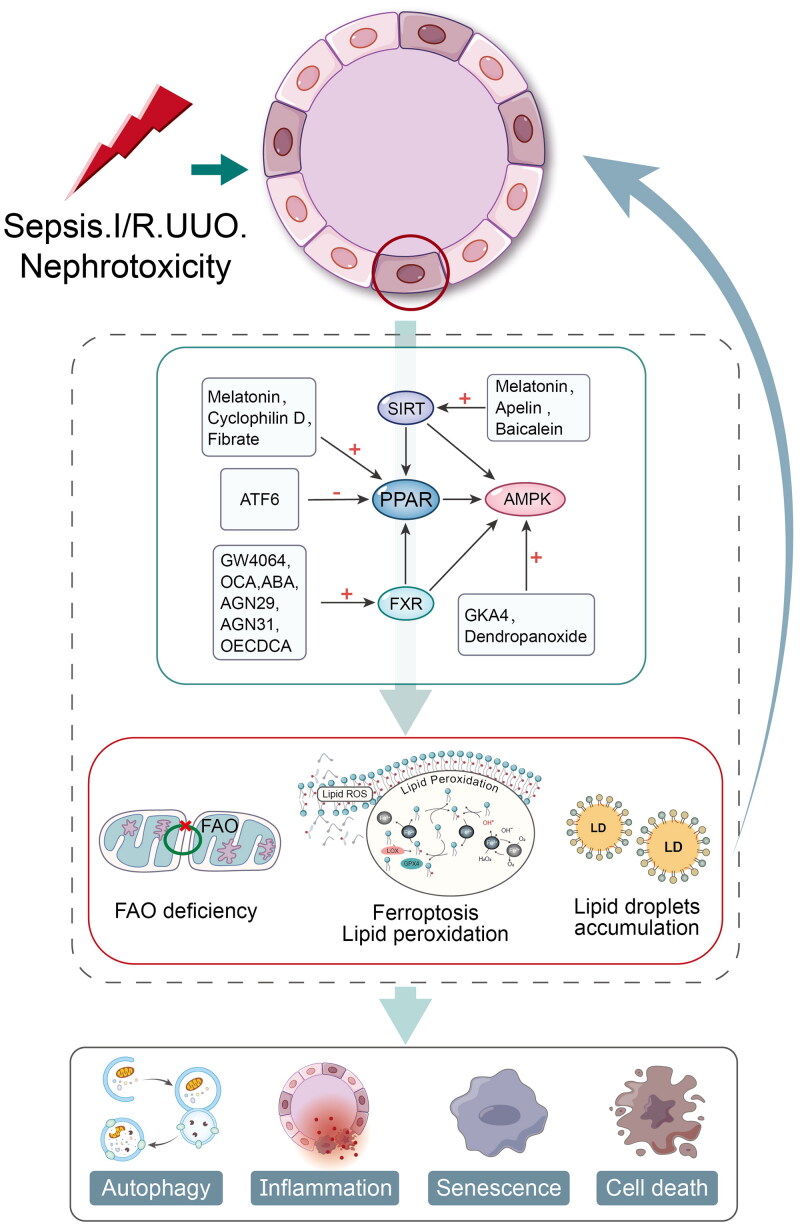

Lipid droplet aggregation, lipid peroxidation, ferroptosis, mitochondrial damage, and compromised fatty acid oxidation (FAO) within renal tubular cells occur during acute kidney injury. These impairments, in turn, intensify AKI, creating a vicious negative feedback loop. Lipid metabolism is regulated through the PPAR, SIRT, FXR, and AMPK pathways, which affect mitochondrial function, FAO, lipid peroxidation, ferroptosis, and lipid droplet formation. Red font represents the agonists of drug-related pathways.

## 1. Introduction

AKI has a high incidence and a high mortality rate. While the prognosis of AKI patients has improved to a certain extent, the disease can stem from various factors, and the underlying pathological mechanisms are intricate. Approximately, 30% of AKI cases progress to chronic kidney disease (CKD), and AKI represents an independent risk factor for CKD [1,2]. Therefore, timely detection and intervention are highly important for improving patient outcomes. Proximal tubules require a substantial amount of energy to maintain renal function, and fatty acid oxidation (FAO) is the pivotal source of energy [3,4]. Lipid metabolism plays a crucial role in AKI development [5]. Therefore, comprehending the variations in lipid metabolism and regulatory elements during AKI progression could present novel avenues for the timely detection and management of AKI. Consequently, unraveling the modifications in lipid metabolism and regulatory factors throughout the progression of AKI may provide new perspectives on the early recognition and mitigation of this condition.

Lipids play a significant role in the energy metabolism and signal transduction pathways in the organism and make up biological membranes. The lipid family comprises fatty acids, triglycerides, phospholipids, sphingolipids, sterol lipids, and other constituents. In addition, various regulatory pathways are involved in the pathological process of AKI [6]. Tubular epithelial cells (TECs) rely heavily on FAO to obtain energy. Metabolic transformation in renal pathology is a complex process. An exploratory metabolomic analysis based on ultrahigh-performance liquid chromatography–quadrupole time-of-flight mass spectrometry of HK-2 cells under hypoxia–reoxygenation conditions revealed that lipids and lipid-like molecules accounted for the majority of the changed metabolites [7]. Thus, understanding the changes in lipid metabolism and its regulatory factors in AKI is crucial.

## 2. Disordered lipid metabolism in AKI

Various previous studies have employed techniques such as liquid chromatography–mass spectrometry [8], lipidomics [9], proteomics [10], and transcriptomics [11] to demonstrate disturbances in the lipid composition and metabolism in AKI patients or animal models. These disruptions include defects in FAO, lipid droplet formation, membrane phospholipid alterations, particularly sphingolipid conversion, and fatty acid alterations. Using lipid metabolism products as biomarkers could enable the early detection of AKI and aid in assessing its severity. Early intervention to correct abnormalities in lipid metabolism can mitigate AKI damage and improve patient prognosis.

### 2.1. Fatty acid oxidation

The impairment of TECs is a pivotal stage in the development of AKI. Under normal physiological conditions, FAO serves as the primary energy source for TECs. However, various factors that induce AKI, such as ischemia–reperfusion (IR), cisplatin, sepsis, rhabdomyolysis, acidosis, folic acid, and kanamycin, commonly lead to functional impairment in FAO. This deficiency in FAO is closely associated with mitochondrial dysfunction [12], reduced activity of enzymes involved in lipid metabolism, and dysregulation of upstream pathways.

Mitochondria act as the principal location for FAO. As a result, disturbances in mitochondrial function cause partial FAO deficiencies, hinder mitochondrial biosynthesis, promote mitophagy, and affect mitochondrial dynamics [13]. Mitochondrial impairment is observed in mouse models of cisplatin- and IR-provoked AKI [14,15], and it further amplifies impairments in FAO, diminishes adenosine triphosphate synthesis, and increases the accumulation of reactive oxygen species, thus causing a detrimental cycle.

The diminished enzymatic function of FAO is both a prominent cause and an indicator of FAO abnormalities. In models of cisplatin-induced AKI, the expression of key enzymes involved in fatty acid metabolism, namely, carnitine palmitoyltransferase 1 (CPT1), long-chain acyl-CoA dehydrogenase (ACADL, also known as LCAD), and medium-chain acyl-CoA dehydrogenase (MCAD), is notably reduced [12,16]. The restoration of CPT1 expression was shown to substantially ameliorate renal injury [17]. Similar alterations have been noted in the context of ischemia–reperfusion injury (IRI) as well as in models of sepsis-induced AKI [18,19]. Proteomic analyses revealed a notable reduction in the expression of Acox1 and Crot, enzymes relevant to FAO, on the third and seventh days in IRI mice, with the extent of the reduction being associated with the injury score [10]. The enrichment of proteins associated with FAO provides protection against cisplatin-induced acute kidney injury (AKI) [20]. Furthermore, several signaling pathways, such as the peroxisome proliferator-activated receptor (PPAR), adenosine 5′-monophosphate (AMP)-activated protein kinase (AMPK), sirtuin (SIRT), hypoxia-inducible factor, and transforming growth factor-β1 pathways, are crucial in the regulation of FAO in AKI [21].

Deficiencies in FAO result in inadequate energy substrates for the subsequent tricarboxylic acid cycle, leading to reduced ATP generation. This, in turn, hinders the assembly of cytoskeletal proteins and causes the depletion of the brush border, as well as the detachment of TECs [22,23]. The impairment caused by TECs leads to a decline in kidney function and subsequently initiates fibrosis. In models of IR, the suppression of peripheral cellular FAO additionally accelerates the progression from AKI to CKD [21,24,25]. By modulating FAO-related genes, enhancing mitochondrial function, and modifying the activation of upstream signaling pathways in FAO metabolism, it is possible to increase FAO and consequently alleviate AKI.

Notably, there is a marked increase in FAO during the initial stage of AKI [26], as evidenced by the increase in the FAO score 6 h after unilateral IRI, which indicates increased lipid metabolism in injured type 1 proximal tubule cells during the early phase [11]. In addition, metabolomics of protein metabolism revealed a compensatory impact of the noninjured contralateral kidney in patients with unilateral IRI. Increases in the fatty acid signaling pathway activity, lipid levels, and fatty acid levels were observed at 4- and 24-h time points postreperfusion [27].

During the postrecovery phase of AKI, the patterns of FAO vary across different models. For example, in a unilateral IR-induced AKI model, renal tubular FAO gradually recovered over time. The expression of key enzymes involved in FAO tended to reach normal levels by the 21st day. Conversely, in a model of unilateral ureteral obstruction (UUO), Acox1 expression continued to decrease in renal tubular cells, and persistent lipid droplet accumulation in the epithelial region was observed even after 21 days. These findings suggest that the restoration of FAO might be linked to the severity and nature of the injury and be associated with interstitial fibrosis and the transition to CKD [10,28].

During the acute phase of AKI, we observed a compensatory increase in FAO when hypoxia persisted. However, this adaptive mechanism transforms into a deficiency. In the later stages of recovery, certain renal tubules regain their ability to utilize FAO for energy production, whereas others are influenced by unidentified factors and continue to exhibit low FAO, which results in lipid buildup and a potential harm. This lipid accumulation could contribute to chronic inflammation, renal fibrosis, and unfavorable outcomes of AKI. Notably, there are two pivotal turning points to consider: the first turning point is the transition from compensatory FAO acceleration to deficiency, and the second turning point is the attainment or persistence of FAO deficiency. Investigating the timing and mechanisms of these two turning points may provide a deeper comprehension of the metabolic alterations occurring in renal tubules during AKI.

### 2.2. Lipid droplets and lipid accumulation

AKI is distinguished by the notable accumulation of lipids, which is strongly correlated with the severity of renal injury. Although triglycerides themselves are deemed nonharmful, the excessive burden of free fatty acids (FFAs) and subsequent metabolites is detrimental [29,30]. The etiologies of lipid excess in various renal disorders vary; however, it typically arises from the dysregulation of lipid uptake, lipid biosynthesis, FAO, and lipid efflux [5]. In IRI, renal accumulation of cholesterol, phospholipids, and sphingolipids is observed [26]. In another model of AKI, induced by metabolic acidosis, elevations in phospholipid and sphingolipid levels were also observed via liquid chromatography–mass spectrometry [8]. In a sepsis group, a significant increase in the number of lipid droplets was observed 12 h after surgery, and the number reached a maximum at 18 h postsurgery [31]. In both the IR and UUO models, damaged TECs exhibited upregulation of genes associated with the formation of lipid droplets [11]. The excessive accumulation of lipids leads to lipotoxicity, which is believed to play a role in the progression of AKI [5]. Reducing lipid accumulation can alleviate tubular inflammation and cellular apoptosis [32], as well as mitigate renal damage and the progression of CKD [33]. A histone methyltransferase promotes AKI by inhibiting lipase carboxylesterase 1 (Ces1) and increasing lipid accumulation [34]. Furthermore, upregulation of uncoupling protein 1 can reduce lipid accumulation and provide relief from AKI [35], thereby offering a novel approach to AKI treatment.

Nevertheless, it is worth mentioning that there are variations in the genesis, apex, and dissipation of lipid droplets across various models. In a single iri6h model, there was a notable increase in the number of lipid droplets, which, however, became imperceptible on the seventh day. Conversely, in the UUO model, an initial absence of lipid droplet aggregation was observed, yet by the 10th day, tubular lipids accumulated [11].

### 2.3. Alterations in the lipid composition

#### 2.3.1. Phospholipids

Phospholipids, as the primary constituents of biological membranes, undergo frequent alterations in AKI, reflecting the severity of the condition. Decreases in the phosphatidylethanolamine/phosphatidylinositol (PE/PI) and phosphatidylethanolamine/phosphatidylcholine (PE/PC) ratios in the kidneys of AKI patients signify shifts in the composition of biological membranes, which results in changes in membrane stability [36]. In folate-induced AKI, the levels of PE and hemolytic sulfatide substances decrease, whereas those of phospholipid species, such as PI, PC, and phosphatidylglycerol, increase. Notably, the distribution of phospholipid species in different kidney regions may be associated with renal function. For example, the abundance of medullary PE species is inversely related to renal function, whereas PI species are positively correlated with the loss of renal function across all regions [37]. Similar changes in the phospholipid composition were observed in cisplatin- and IR-induced AKI, with a reported decrease in the PC content in cisplatin-induced AKI [9,38], together with a significant loss of membrane lipids [20]. Following treatment with realgar (an arsenic-containing traditional Chinese medicine), increased levels of hemolytic phospholipids, including lysophosphatidylcholine (LPC) and lysophosphatidylethanolamine (LPE), were observed [39]. LPC directly or indirectly triggers the generation of reactive oxygen species, resulting in cellular oxidative damage [40]. Variations in phospholipid metabolism play critical roles in cisplatin- and lipopolysaccharide (LPS)-induced kidney injury [41,42].

We observed notable alterations in phospholipid species in AKI patients and differences among various types of AKI models. The variations in phospholipid species in cortical and medullary regions also exhibit differences, potentially corresponding to the severity of kidney diseases. Therefore, measurement of specific phospholipid contents may aid in the early detection and categorization of AKI. Interventions targeting phospholipid transformations during AKI may contribute to ameliorating this condition, thus offering a potential avenue for early identification of and therapeutic intervention in AKI.

#### 2.3.2. Sphingolipids

Sphingolipids and glycerophospholipids are closely interlinked, forming a complex relationship. Ceramides, which are intermediate products of sphingolipids, have been identified as lipotoxins that contribute to the pathogenesis of various forms of AKI [43,44]. Studies have revealed excessive accumulation of sphingolipids, particularly ceramides, in renal tissues during IRI- and cisplatin-induced AKI [45]. This accumulation is attributed primarily to overactivation of the sphingolipid synthesis pathway in response to tubular injury stress, resulting in sphingolipid-mediated mitochondrial autophagic damage and tubular cell apoptosis. The inhibition of sphingolipid generation was shown to protect mice from cisplatin-induced AKI [44].

Intermediate products of sphingolipid metabolism also play critical roles in AKI [46], and the inhibition of glucosylceramide synthase exacerbates cisplatin- and glycerol-induced AKI [47,48]. In the context of rhabdomyolysis- and cephaloridine-induced AKI, triacylceramide promotes albumin absorption in tubules, thereby impeding preventive measures against AKI [49]. Neutral ceramidase, an enzyme responsible for converting ceramides into sphingosine, has been shown to alleviate the development of cisplatin-induced AKI by increasing autophagy and inhibiting cell death [50], thus resulting in improved renal function. Sphingosine-1-phosphate receptor 1 (S1PR1) has been demonstrated to mitigate cisplatin-induced AKI by reducing mitochondrial dysfunction [51]. Sphingosine-1-phosphate can activate rho-kinase, causing tubular damage [52]. Sphingosine also induces injury to proximal tubule cells *in vitro* [53], and intervention therapy has a protective effect against AKI by promoting the proliferation of renal epithelial cells after IRI and limiting a crosstalk between endothelial and immune cells during injury [54]. The binding of sphingosine-1-phosphate to its receptor S1PR1 has a protective effect against AKI [55].

The excessive accumulation of sphingolipids and the disruption of sphingolipid metabolism during AKI contribute to sphingolipid-mediated mitochondrial autophagic damage and apoptosis, exacerbating AKI progression. Therefore, early intervention to restore the physiological conditions of sphingolipid metabolism may alleviate AKI.

#### 2.3.3. Fatty acids

In patients with AKI, a surge in the levels of FFAs has been observed. Notably, there are significant spikes in the levels of saturated fatty acids, such as palmitic acid (PA) and stearic acid, approximately four hours following ischemic injury [27]. Studies have shown that lowering renal fatty acid levels can lead to an improvement in IR injury [56]. Concurrently, an enrichment of unsaturated fatty acids has also been noted in heatstroke-associated AKI [57]. The regulation of TECs in different fatty acid environments is critically important. Research suggests that PA triggers the redistribution of phospholipids in renal tubular cells [58], which in turn leads to increased nonphysiological autophagic degradation of phospholipids and extensive autophagy in biological membranes. This ultimately results in the accumulation of substantial amounts of phospholipids in lysosomes, inadequate acidification, impaired physiological autophagic function, and an impact on lysosomal function [32]. PA-induced cytoplasmic changes are accompanied by increases in the levels of neurotoxic sphingolipids and diacylglycerol, which promote endoplasmic reticulum stress and ferroptosis [59].

Eicosapentaenoic acid (EPA), a 20-carbon omega-3 fatty acid, has the potential to influence lipid metabolism, cell signal transduction, and gene expression [60]. EPA has been found to mitigate IR-induced renal fatty acid toxicity by relieving lysosomal dysfunction and promoting the transfer of fatty acids from lipid droplets to mitochondria for β-oxidation. This helps alleviate mitochondrial dysfunction, inflammation, fibrosis, oxidative damage, and apoptosis, thus mitigating IR-induced AKI [32,61]. Docosahexaenoic acid (DHA) has similar effects. Monounsaturated oleic acid reduces the levels of FFAs, thereby inhibiting cell apoptosis and ferroptosis [62,63]. However, notably, the effects of unsaturated fatty acids on AKI are specific to the type of AKI and the mechanism of injury. Dietary supplementation with DHA alleviates renal tubular injury and kidney damage in rhabdomyolysis-induced AKI but exacerbates kidney damage in FA-induced AKI mice [64].

AKI is characterized by substantial changes in fatty acids. An increase in the levels of fatty acids exacerbates renal damage by impacting endoplasmic reticulum oxidative stress, reducing autophagy, and inducing ferroptosis. The effects of unsaturated fatty acid supplementation on AKI are bidirectional and may depend on whether the injury mechanism involves ferroptosis [64]. Furthermore, straightforward dietary fatty acid supplementation does not alleviate the damage to kidney function [65]. Therefore, dietary fatty acid supplementation can serve only as nutritional support therapy, and the type of AKI must be considered. Nonetheless, reducing fatty acid toxicity remains a potential therapeutic approach for treating AKI.

## 3. Lipid metabolism disorders promote AKI

The disruption of lipid metabolism during AKI contributes to the progression of this condition. Early intervention by ­regulating lipid metabolism can mitigate the damage caused by AKI. For example, myoinositol oxygenase moderates cephaloridine-induced AKI by activating arachidonate 12-lipoxy­genase and the generation of 12-hydroxyeicosatetraenoic acid [66], and *Pediococcus acidilactici* GKA4 was shown to alleviate cisplatin-induced AKI through the phosphatidylinositol 3-kinase–protein kinase B axis [67]. Aberrant lipid metabolism can adversely impact the prognosis of AKI by triggering ferroptosis and inflammatory responses and facilitating the transition from AKI to CKD.

### 3.1. Lipid peroxidation promotes AKI

Lipid peroxidation is a deleterious process that directly causes harm to membrane structures or indirectly releases toxic byproducts [68], which results in severe vasoconstriction and oxidative damage [69]. Reactive isolevuglandins (IsoLGs), resulting from the peroxidation of arachidonic acid (AA), cause mitochondrial dysfunction and inflammation, leading to detrimental effects. A reduction in the IsoLG levels could decrease the mortality rate in an LPS-induced AKI model [70]. AKI injury could be improved by decreasing mitochondrial lipid peroxidation [71]. Ferroptosis, which is characterized by iron-dependent accumulation of lethal levels of lipid peroxides, is induced by renal IR and leads to the production of reactive oxygen species in renal TECs through the activation of the aryl hydrocarbon receptor [72]. Lipidomic analysis has indicated that PEs containing AA or adrenic acid are critical membrane phospholipids involved in lipid peroxidation, which drives ferroptosis [73]. Conversely, cells supplemented with AA or other polyunsaturated fatty acids are susceptible to ferroptosis [74]. Alleviating lipid peroxidation and ferroptosis can mitigate the progression of AKI [75]. Ferroptosis is widely recognized to play a crucial role in AKI, and drugs that target ferroptosis and exert protective effects on the kidneys have been developed [76]. In conclusion, the disruption of lipid metabolism during AKI results in lipid peroxidation and ferroptosis, further exacerbating AKI damage, which highlights the essential need for early intervention to mitigate AKI damage.

### 3.2. High levels of fatty acids promote AKI

Lipid metabolism and immune cell function are closely intertwined. Prior administration of monophosphoryl lipid A diminishes detrimental proinflammatory responses and enhances kidney function during sepsis [77]. Elevated FFA levels can activate immune cells, thereby promoting the production of proinflammatory cytokines and contributing to AKI [78,79]. Trimetazidine notably reduces myeloperoxidase expression and FFA-induced inflammatory cytokine levels, mitigating folic acid-induced AKI [80]. Moreover, the excessive imbalance of lipid metabolism products, particularly AA and its derivatives, has been extensively discussed in the context of the inflammatory process of AKI [81]. When renal neutrophils are recruited, 20-hydroxyeicosatetraenoic acid (20-HETE) exacerbates AKI-related inflammation [82]. Lipids generated by cytochrome P450 (CYP) enzymes can decrease kidney inflammation, alleviate IRI, and prevent cyclosporine-induced renal dysfunction [83,84]. Treprostinil, a prostacyclin analog, ameliorates renal IRI [85], but the combined effects of energy damage and prostaglandin metabolism may induce renal tubular dysfunction or damage and stimulate inflammatory responses [86].

In addition, treatment with PA induces the activation of the cyclic guanosine monophosphate-adenosine monophosphate synthase (cGAS) signaling pathway [87,88], promoting AKI inflammation. Free cholesterol influences the involvement of β-glucosylceramide, an endogenous Mincle ligand, in renal tubular inflammation after renal IRI [89]. Disruptions in glycerophospholipid metabolism exacerbate cisplatin-induced kidney injury through nuclear factor kappa-B activation [90]. Inhibition of Lyso-Sulf, a unique lipid class derived from essential fatty acids, significantly reduces monocyte differentiation into macrophages [91], indicating its anti-inflammatory properties.

Specialized proresolving mediators derived from a specific class of essential fatty acids play a vital role in resolving inflammation. For example, lipoxin D2 can diminish LPS-mediated kidney injury [92], and metabolites of DHA can inhibit endotoxin-induced NF-κB activation and AKI pathogenesis [61]. In mice, the administration of other metabolites before IR was shown to reduce kidney dysfunction and morphological damage [93].

The interplay between lipid metabolism and immune inflammation drives pathological injury in AKI. Modulating lipid metabolism may alleviate renal inflammation, while immune responses can influence lipid metabolism. Identifying key targets in this interplay and breaking the relentless cycle between abnormal lipid metabolism and AKI is advantageous. Thus, modulating the interaction between lipid metabolism and the immune system to alleviate kidney injury is feasible.

In patients with AKI, renal lipid metabolism is significantly impacted. Reciprocally, disturbances in lipid metabolism can exacerbate the progression of AKI by increasing renal inflammation, lipid peroxidation, and ferroptosis. Therefore, a comprehensive exploration of lipid metabolism during AKI and alleviation of lipid metabolism disruptions to mitigate AKI and interrupt the relentless cycle of abnormal lipid metabolism and AKI represent a promising intervention direction for AKI treatment. Currently, several compounds that alleviate lipid-related damage have been evaluated in preclinical models of AKI and yielded favorable observational results [94].

## 4. Regulation of lipid metabolism in AKI

Lipid metabolism in AKI is controlled by numerous extensively investigated upstream regulatory pathways. These pathways are intricate in nature and intricately interact with one another. In addition to governing lipid metabolism, these pathways assume distinctive functions in regulating oxidative stress, inflammation, and cellular survival. Hence, a thorough understanding of their mechanisms and appropriate interventions hold promise for the therapy of AKI (Table 1).

**Table 1.** Regulation of lipid metabolism.

AKI model	Molecule	Outcome	Target	Reference
Cisplatin PTC and mice	Sanglifehrin A	Increasing FAO Mitochondrial damage Lipid accumulation Tubular damage Neutrophil accumulation Tubular cell cycle arrest	Inhibits mitochondrial interaction between cyclophilin D and PPARα	[95]
Cisplatin PTC and mice	GW4064	Increasing FAO Reducing lipid accumulation Ameliorating renal functional and histological damage Anti-inflammation Antiapoptosis	Activation of FXR, PPAR, and PGC	[18]
Cisplatin PTC and LLC-PK1 cells	fibrate	Anti-inflammation Antiapoptosis	Activation of PPAR	[96]
AAI PTC and mice	KLF15 knockdown	Reducing FAO Exacerbating injury and fibrosis	Loss of KLF15 and downregulating PPAR	[97]
Iohexol-treated mice and NRK-52E cells	Melatonin	Antioxidative stress Antiapoptosis Anti-inflammation	Activation of SIRT3	[98]
Cisplatin mice and TECs	Melatonin	Increasing FAO Antiapoptotic properties Reducing the number of lipid droplets	Upregulating PPARα	[99]
Cisplatin-PTC and mice	Apelin	Protecting mitochondria Downregulating MFF Upregulating OPA1	Activation of SIRT3	[100]
PMB-induced mice and HK-2 cells	Baicalein	Inhibits PMB-induced ferroptosis	Activation of SIRT1	[101]
IRI mice and HK-2 cells	ATF6	Deranges fatty acid metabolism Apoptosis Promotes tubulointerstitial fibrosis	Downregulated PPARα	[102]
IRI mice	G9a	Increasing renal lipid accumulation Impairing TECs	Competitively binds to the promoter region of Ces1 with FXR	[34]

PTC: proximal tubular cell; HK2: human kidney-2; KLF15: Kruppel-like factor 15; PMB: polymyxin B; ATF6: activating transcription factor 6; G9a: known as EHMT2, a histone methyltransferase; MFF: mitochondrial fission factor gene; OPA1: optic atrophy 1; PPARs: peroxisome proliferators-activated receptors; PGC: peroxisome proliferator-activated receptor-gamma coactivator; SIRTs: sirtuins; FXR: farnesoid X receptor; Ces1: carboxylesterase 1.

### 4.1. Peroxisome proliferator-activated receptor

PPAR is a critical ligand-activated transcription factor of utmost importance in the regulation of lipid metabolism [103]. It plays a central and essential role in governing fatty acid transport, synthesis, and oxidation. In experimental models involving cisplatin-, aristolochic acid-, and IR-induced kidney injury, notable decreases in PPARα expression were observed [104]. These decreases were accompanied by diminished cellular ATP production, compromised intracellular FAO, impaired fatty acid transport, and the aggregation of lipid droplets in the kidneys. Conversely, these dysregulated functions were ameliorated through the restoration of PPARα activity by using bezafibrate or alternative methods [96,105]. Furthermore, the severity of kidney injury was found to be correlated with PPAR deficiency, as evidenced by the fact that children with sepsis displayed more extensive renal damage than those without sepsis, which was accompanied by the inhibition of PPARα-associated whole-genome expression profiles [106].

The PPAR lipid metabolism pathway is also influenced by upstream factors. In mice with UUO, increased expression of signal transducer and activator of transcription 6 facilitated renal lipid accumulation by repressing PPARα and its target genes associated with FAO [107]. However, PPARα exerts an opposite effect on cisplatin-induced AKI [104]. Moreover, factors such as Krüppel-like factor 15, proximal tubule angiotensin D, and membrane-associated protein A1 modulate renal FAO by regulating PPARα [95,97,108]. In contrast, a PPAR inhibitor that activates transcription factor 6 exacerbates kidney damage [102].

Peroxisome proliferator-activated receptor-gamma coactivator (PGC)-1α, which facilitates the generation of new mitochondria, is directly or indirectly controlled by PPAR. In mouse models of sepsis- and IR-induced AKI, the expression of PGC-1α had a similarly decreasing pattern as that of PPAR. The insufficiency of PGC-1α resulted in a diminished mitochondrial abundance, reduced ATP production, and a corresponding exacerbation of kidney damage [109,110]. In contrast, the overexpression of PGC-1α in renal tubule cells ameliorated lipid accumulation and tubular injury [111]. PGC-1α can alleviate IR-induced AKI by mitigating mitochondrial dysfunction through the p38 signaling pathway [112].

### 4.2. Adenosine monophosphate-activated protein kinase

AMPK also plays a crucial role in AKI and lipid metabolism, specifically through the AMPK/acetyl-CoA carboxylase pathway. Acetyl-CoA carboxylase, a pivotal enzyme in fatty acid synthesis, is inhibited through AMPK activation. This inhibition enhances CPT1 activity, promotes FAO, suppresses fatty acid synthesis, and thereby mitigates AKI damage [113]. AMPK-mediated autophagy and inflammation have been identified as important regulatory mechanisms in IR-induced AKI [114]. The targeting of AMPK enhances IR-induced tubular injury and apoptosis, which suggests a promising pharmacological target for the prevention and treatment of AKI.

### 4.3. Farnesoid X receptor

FXR, which was previously believed to alleviate IR-, LPS-, or cisplatin-induced AKI through pathways involving inflammation, oxidative stress, and apoptosis, has recently been found to regulate lipid metabolism [115]. FXR influences ferroptosis by modulating the expression of genes such as fatty acid-binding protein and the apoptosis-inducing factor mitochondria associated 2 [116]. FXR governs the transport of fatty acids and initiates the breakdown of lipids via Ces1 [34]. Furthermore, it can mitigate lipogenesis [117]. FXR also interacts with pathways such as the PPAR and AMPK pathways. Treatment with FXR agonists has been shown to mitigate renal damage during AKI by downregulating genes associated with fatty acid synthesis and upregulating those involved in FAO and lipid breakdown [18,118]. Interestingly, FXR activation has been shown to impede the progression of AKI into CKD, which may be linked to the restoration of FAO. Although FXR antagonists and agonists have entered clinical trials, their focus has predominantly been on liver diseases, while clinical trials specifically targeting kidney diseases, particularly AKI, have yet to be conducted [118].

### 4.4. Sirtuin

Sirtuin deacetylases play critical roles in maintaining mitochondrial function and lipid metabolism, thereby exerting an indispensable influence on various subtypes of AKI. An exemplary example includes the activation of SIRT 3, which enhances mitochondrial function through the deacetylation of liver kinase B1, consequently leading to the activation of AMPK, PPAR, and other signaling cascades. These activities lead to strengthening FAO, diminishing ROS production and lipid peroxidation, and mitigating the detrimental effects of ferroptosis to ultimately improve the outcomes of AKI. Notably, the activation of SIRT 3 has been observed as a result of melatonin, resveratrol, and apelin treatment [98,100,104].

Conversely, the suppression of SIRT 1 in mice that were subjected to cecal ligation and puncture curtailed FAO while simultaneously increasing the mortality rate [119]. Quercetin, through its effect on deacetylation of SIRT 1/p53, inhibits ferroptosis and enhances amphotericin B-induced AKI [101]. Furthermore, SIRT6 has been implicated in regulating kidney lipid accumulation, inflammation, and fibrosis and maintaining mitochondrial quality control [120].

The aforementioned regulatory pathways are complex, with multiple molecules interacting with each other. Notably, lipid metabolism regulation extends beyond these pathways. Research indicates that miR-21-3p acts as a regulator of lipid metabolism in AKI by modulating forkhead box O1 levels in TECs [31]. Targeted inhibition of forkhead box O1 was shown to decrease lipid droplet accumulation and improve kidney damage in septic mice. Additionally, hypoxia or nutrient deficiency results in the dephosphorylation of phosphatidic acid phosphohydrolase 1, which subsequently leads to a decrease in PA, PC, and phosphatidylserine (PS) levels and limits the transfer of PS to the mitochondrial inner membrane, ultimately preventing the accumulation of PE [121]. The hypoxia-inducible factor and transforming growth factor-β1 pathways are also associated with lipid metabolism regulation in AKI [21]. Undeniably, lipid metabolism regulation is a complex process that warrants extensive and in-depth research to establish a comprehensive regulatory network.

## 5. Clinical translation

Lipidomic analysis, which serves as an early indicator of lipid levels in blood and urine, has the potential for diagnosing and assessing critical illnesses. Recent studies have revealed that variations in the CYP-like AA pathway may increase the likelihood of developing AKI, and targeted drug interventions aimed at this pathway may provide novel preventive measures for AKI [122], but clinical research on CYP in AKI is currently very limited, and the side effects and individual differences associated with CYP cannot be ignored. These still require further exploration. Genetic polymorphisms in high-density lipoprotein are closely linked to increased susceptibility to AKI during sepsis [123,124]. Research suggests that reduced serum levels of ApoA5 may serve as a prognostic marker for pediatric sepsis-induced AKI [125].

Liver fatty acid-binding protein (L-FABP), a member of the lipid-binding protein superfamily, is integral to the cellular process of shuttling fatty acids across the extracellular and intracellular membranes. In a seminal study, Susantitaphong et al. validated urinary L-FABP as a biomarker for the diagnosis of AKI, achieving a diagnostic sensitivity of 74.5% and a specificity of 77.6% [126]. To further corroborate its utility, Noiri et al. demonstrated that urinary L-FABP levels, with a significant increase detectable within the first hour following ischemic insult, surpass those of blood urea nitrogen and urinary N-acetyl-β-d-glucosaminidase for the diagnosis of AKI across diverse animal models [127].

Although animal and *in vitro* experimentation has demonstrated the potential of regulating lipid metabolism to mitigate AKI damage and improve outcomes, clinical investigations in this field remain limited. Successful clinical translation necessitates careful consideration of the drug dosage, administration method, timing, safety, specificity, side effects, individual variances, and drug resistance. Furthermore, further exploration is needed to strike a balance between the body’s adaptability and injury when the optimal timing for intervention is determined.

## 6. Conclusions

The metabolic alterations during AKI are exceedingly intricate, encompassing both FAO and glycolysis, and result in bidirectional, time-dependent effects during physiological stress, which necessitates prompt intervention. Reestablishing normal FAO is linked to fibrosis and CKD, whereas comprehending two pivotal inflection points may facilitate a deeper understanding of metabolic changes in renal tubules during AKI, thereby guiding therapeutic approaches. Targeted interventions at distinct sites may confer more advantageous outcomes of AKI treatment. Furthermore, additional research is needed to investigate the metabolic changes in cells other than those of renal tubules within the kidney. Vicious cycles ensue at interaction junctures, including the interplay between AKI and lipid modifications, mitochondrial function, and FAO, as well as between lipid metabolism and immune inflammation. Recognizing the instigation points of harm and its timely cessation are vital in minimizing cascading injury.

## Supplementary Material

Figure 1.jpg
