# Supplementary figures and images for: Exploring the role and therapeutic potential of lipid metabolism in acute kidney injury
Source: Ren Fail. 2024 Sep 25;46(2):2403652. doi: 10.1080/0886022X.2024.2403652 (PMC11425701; doi:10.1080/0886022X.2024.2403652)

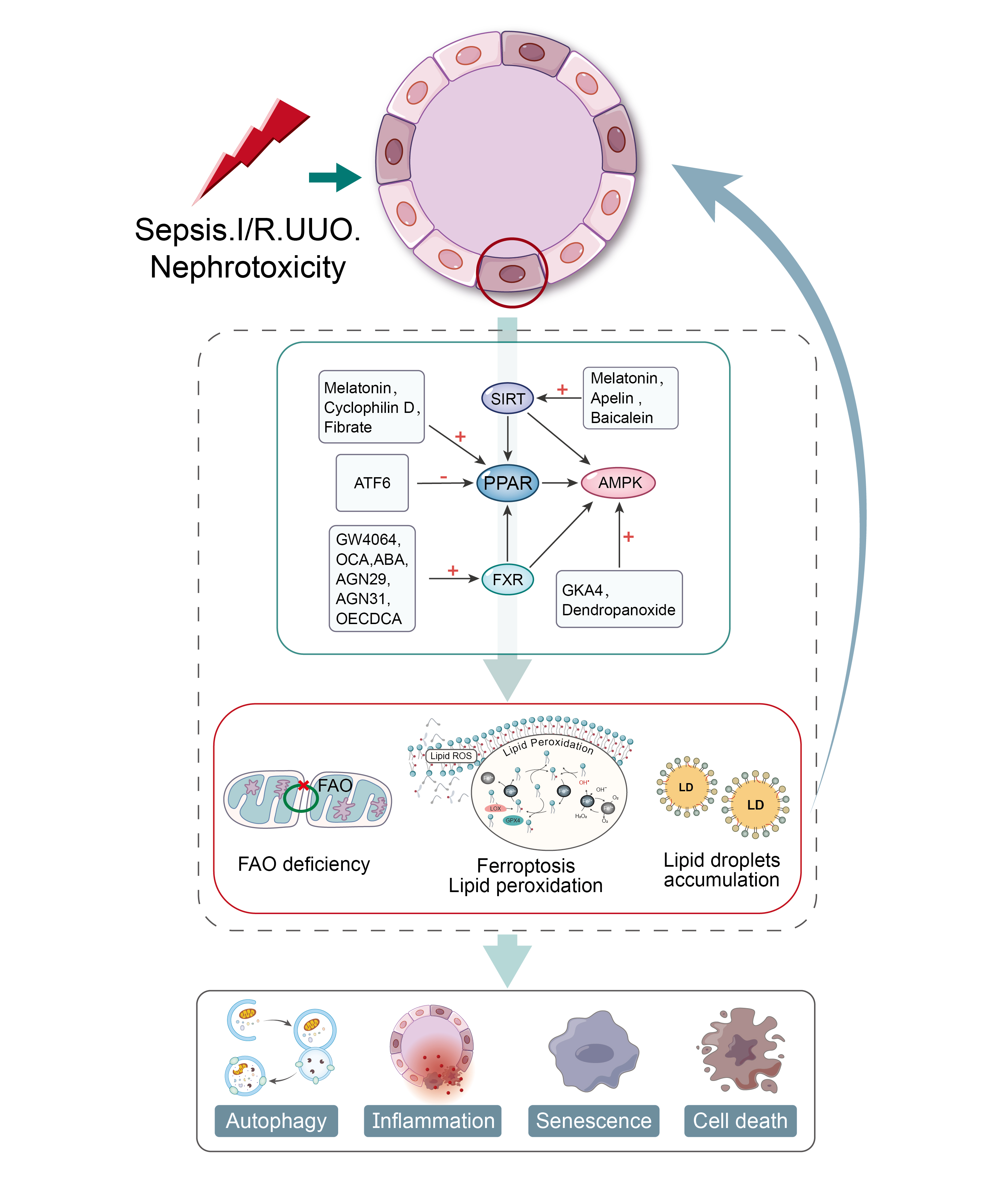

Supplement: Figure 1.jpg [file IRNF_A_2403652_SM1152.jpg]
